# Supplementary material for: Myocardial extracellular volume quantification by computed tomography predicts outcomes in patients with severe aortic stenosis
Source: PLoS One. 2021 Mar 10;16(3):e0248306. doi: 10.1371/journal.pone.0248306 (PMC7946277; doi:10.1371/journal.pone.0248306)
Supplement: Attachment — Submitted filename: response to reviewers3.docx [file pone.0248306.s002.docx]

**Editor**

**Comment**: *minor occurrence of overlapping text.*

Response:

1. For "Staging classification of aortic stenosis based on the extent of cardiac damage" by Phillip Genereux et al – As far as we noticed, the overlapping text is the definitions of the different stages of AS. These definitions are clear and cannot be changed. This overlap appears only in the methods section and is backed up with an appropriate reference. **Please let us know if there are any overlaps with this paper or if the editor would like us to change it somehow.**
2. For "Measurement of Myocardial Extracellular Volume Fraction by Using Equilibrium Contrast-enhanced CT: Validation against Histologic Findings" by Steve Bandula et al **–** Unfortunately we could not identify the overlap between this paper and our paper. **We would highly appreciate your guidance regarding what are the exact parts of our paper which overlapped, so we could address it.**

**Reviewer 1:**

Thank you for your helpful comments and suggestions. Below please find the responses that were incorporated into the manuscript.

**Comment: "***ECV could be evaluated in only 75 out of 85 AS patients initially enrolled, due to inadequate CT image quality. Methods and results of image quality assessment should be explained*"

**Response**: We thank the reviewer for this comment. In order to make it clear we rephrased the 1^st^ paragraph of the methods section, and added the following text:

"While initially recruited 85 patients with symptomatic severe AS, 10 patients were excluded due to pacemakers, implantable cardioverter defibrillators, metallic foreign objects in the proximity of the heart, and surgical aortic valve replacement. All of these may lead to beam-hardening artifacts, which may degrade the inaccuracy in the ECV measurements". (**Materials and methods section, study cohort subsection, page 5**)

**Comment: "***Both pre-contrast and delayed-phase CT appears to be obtained with a standard prospectively-ECG-triggered scan. As has been demonstrated in ref#22, delayed-phase CT may suffer from severe artifact caused by beam-hardening, motion, and partial scan, which may degrade the accuracy of ECV measurement*"

**Response**: We fully agree with the reviewer, therefore 10 patients with metallic devices were excluded from the study cohort in order to make our measurement accurate and reproducible.

**Comment: "***ECV was measured placing a ROI in the septal wall only. Global ECV or at least averaging of ECV of multiple locations, should more reliably represents the overall extent of LV fibrosis*"

**Response**: We thank the reviewer for this comment. Indeed, global ECV calculation is now available with a dedicated software. Measurement in the myocardial septum is an accepted method of calculating ECV. To note, our protocol when drawing ROI was that in cases where ECV measurement in the myocardial septum was felt to be possibly inaccurate (e.g thin septum, beam hardening or streak artifacts), than ROI was drawn at lateral wall. If there was uncertainty about which one has the most accurate ECV, an average of these two was calculated. This sentence was added to the methods section **(Materials and methods, page 7, 2^nd^ paragraph).**

Moreover, ECV is a diffuse process, and thus ECV measurement in different segments of the left ventricle should be similar. The septum, which is usually the thickest segment of the myocardium, was felt to be the most accurate segment for ECV calculation.

**Comment**: "*In AS group, previous MI and revascularizations were common. There is a risk that ECV was measured in infarcted tissue which may not adequately represent the overall fibrosis of the patient. The result of presence and extent of late enhancement should be reported, and discussed in relation to ECV"*

**Response**: We thank the reviewer for this comment. Only 6 patients of the entire cohort had previous MI. The average ECV of those 6 patients is 37%, which is lower compared with the average ECV (40%) in our study cohort. Moreover, none of these patients had an ECV above 46%. Since their average ECV is considered low, we are not concerned with ECV overestimation in those patients. As mentioned earlier in this letter, if the septum appeared to be scarred, or the septum measurement was felt to be inaccurate, ECV measurement was taken at the lateral wall. To note, we carefully avoid ECV measurement in LV segment with myocardial infarction (**Page 7**, **2^nd^ paragraph**).

**Comment**: "*ECV value in patients with AS varied from less than 20% to larger than 70%. Such high ECV indicates that ECV in the myocardium is almost the same as ECV of the blood pool. ECV of 70-80% should be artifactual. ECV of 60-70% is probably due to artifact or presence of infarction".*

**Response**: We thank the reviewer for this comment. We agree that there was a great variation in ECV values among the AS group. However, the great majority of AS patients (80% of them) had an ECV between 26% and 57% (**see results section, page 12, 1^st^ paragraph**). After re-examining our data we found only 3 patients with an ECV between 61 and 70%, and only 1 patient with an ECV >70%. Therefore, those measurements of a very high ECV were not common. For these patients, systolic pulmonary arterial pressure (SPAP) was very high (average 60.3 mmHg for 3 patients, 1 patient did not have SPAP due to lack of TR). We assume that these particular patients had severe diastolic dysfunction, possibly in the setting of extent myocardial fibrosis, which might have resulted in the high ECV values. Although our ECV measurements were made very cautiously, we cannot completely rule out erroneous measurement in those patients. To note, none of these patients had a history of MI, and all had preserved ejection fraction.

**Comment**: "*Presence of infarction that shows high ECV may be the main source of higher rate of adverse events".*

**Response**: Please see above our response regarding MI in our cohort.

**Comment**: "*Wide range of ECV values in normal subject in this study raises suspicion in its accuracy. ECV values in normal subjects (about 26%) has been well established in both CT and MRI as seen in ref#9*".

**Response**: We thank the reviewer for this comment. The mean ECV of the control group in our study was indeed slightly lower compared with the reference above, however all patients but one had an ECV > 15%. Possible explanation for small differences in ECV value between normal subjects in our cohort and the cohort in the cited reference could be related to the age (median age 57 in our cohort compared with 65 in paper by Kurita et al).

Minor points

**Comment**: "*Line 33; “newly-defined” is misleading because it gives an impression that the authors suggested a new classification in this manuscript"*

**Response**: We thank the reviewer for this comment. The text "newly defined" was changed to "recently published" (**see abstract section, line 34**).

**Comment**: "*Line 78 - This sentence is not true anymore*".

**Response**: We cited the new publications suggested by the reviewer, please see **line 77-79**.

**Comment**: "*Line 81; Long-term clinical outcome? With only 12 months of follow up?"*

**Response**: We thank the reviewer for this comment. The phrase long-term was erased (**Introduction section, page 5, 3^rd^ paragraph, Line 82**).

**Comment**: "*Line 101; Scan protocol and reconstruction of pre-contrast and delayed-phase CT should be described in more details. Conversely, CT protocol for coronary CTA can be shortened for this manuscript".*

**Response**: The protocol of the pre- and post-contrast CT scan was described more in details and the contrast-enhanced scan was shortened. **The changes were done in page 6, under CT data acquisition and reconstruction.**

**Comment**: "*Line 103; Acquisition at 75% RR can suffer from severe motion artifact in case of high heart rate. Please report heart rate during acquisition*".

**Response**: Mean heart rate for the study cohort was 72.1 beats per minute (±10.9). This sentence was added to the methods section (**Materials and methods, CT data acquisition and reconstruction, page 6, line 118**).

**Comment**: "*Line 109; Is 50-60ml sufficient for evaluation of delayed enhancement and ECV?"*

**Response**: Our study focused on ECV measurements. The average weight of the study population was 73 kg, which mean that the average contrast agent dose was about 0.8 ml/kg, which is sufficient for ECV calculation mainly in elderly patients with relatively limited GFR. For example, in the study of Abadia et al. (JCCT 2020; 14: 162-167) they used a 70 ml contrast agent despite a higher BMI of their population.

**Comment**: "*Line 126; Representing the patient’s ECV with a ROI drawn on septum only is probably not adequate*".

**Response**: Please see our response to your 3^rd^ major revision comment above.

**Comment**: "*Line 207; Please clarify what the box and whiskers represent".*

**Response**: In order to avoid any confusion, we deleted the ECV mean value in the figure legend, since the middle line in the box plot represent the median value.

**Comment**: "*Line 296; This is not the first study"*

**Response**: We thank the reviewer for this comment. At the time of our initial analysis, we were not aware of such studies. Indeed, in the last few months some studies regarding this issue were published. The text "this is the first study" was changed to "our study is one of very few studies" (**Discussion, page 16, 1^st^ paragraph)**.

**Comment**: "*Line 301; “newly described” is misleading*".

**Response**: We thank the reviewer for this comment. The phrase "newly described" was changed to "recently described" **(Discussion section, page 16, 1^st^ paragraph**)

**Reviewer 2:**

Thank you for your helpful comments and suggestions. Below please find the responses that were incorporated into the manuscript.

**Comment**: "*The aortic interventions were performed in 57 of 75 patients. The prognostic value of CT ECV should be demonstrated by showing Kaplan Meier event-free survival curves in 57 patients who had aortic valve interventions*".

**Response**: Our results were demonstrated as a ROC curve to show the impact of ECV value on the outcomes of the patients independently from valve intervention: TAVI, AVR, and balloon valvuloplasty. The ECV calculation was performed in patients with severe AS undergoing CT before intervention, but not all of them underwent intervention. From a clinical point of view it is crucial to demonstrate our results in this way in order to help the clinician to know which patients would benefit from intervention and which patients would not.

**Comment**: *The staging of AS was successfully performed by echocardiography in only 66 of 75 patients. How were the severity of AS and the indication of aortic valve intervention determined in the patients who enrolled the study?*

**Response:** In our center, severe aortic stenosis is defined as per contemporary valvular heart disease guidelines (symptoms typical of aortic stenosis, mean aortic gradient > 40 mmhg, valve area < 1.0 cm^2^). All patients were examined at our outpatient clinic and then referred to a dedicated consultation 'heart team' forum that includes a multidisciplinary team of clinical cardiologists, imaging specialists, interventional cardiologists, cardiac surgeons, and geriatricians as required. All patients were assessed by trans-thoracic echocardiography and ECG-gated cardiac CT. Information about this process was added to the manuscript (**materials and methods, study cohort, page 6. 2^nd^ paragraph**).

- Baumgartner H, Falk V, Bax JJ, De Bonis M, Hamm C, Holm PJ, et al; ESC Scientific Document Group. 2017 ESC/EACTS Guidelines for the management of valvular heart disease. Eur Heart J. 2017;38(36):2739-91. doi: 10.1093/eurheartj/ehx391

**Comment**: *"Superiority of CT over CMR for the assessment of myocardial ECV fraction, particularly higher spatial resolution, was too much emphasized in the second paragraph of the introduction. In the current study, the ROI was placed in the interventricular septum, indicating that high spatial resolution and SNR improvement by iterative reconstruction are not relevant. Bean gardening artifacts and motion artifacts are major concerns for CT quantification of myocardial ECV fraction*".

**Response**: We thank the reviewer for the comment. We fully agree with the reviewer. We shortened the paragraph and concentrate on the advantages of CT. Iterative reconstruction used by CT and T1 mapping in MRI are not really important for our manuscript and therefore were skipped from the introduction and Ref. 6 and 10 were deleted **(Introduction section, page 4, 2^nd^ paragraph**). The effect of Beam-hardening artifacts was mentioned in the methods section (**page 5, Study cohort subsection**) and limitations section (**page 17, 2^nd^ paragraph**) and this effect was the reason for excluding patients with pacemaker, ICD, AVR or any other metallic objects.

**Comment**: "*The authors emphasized that the implication of ECV fraction using CT has never been studied in patients with severe AS. Please revise the introduction and discussion by citing the following publications*".

**Response**: We thank the reviewer for this comment. At the time of writing this manuscript these studies have not been published yet. These references were incorporated into the manuscript:

- Oda S. Quantification of Myocardial Extracellular Volume With Planning Computed Tomography for Transcatheter Aortic Valve Replacement to Identify Occult Cardiac Amyloidosis in Patients With Severe Aortic Stenosis. Cir Cardiocasc Imaging 2020;13:e010358
  - Tamarappoo B, et al. Prognostic Value of Computed Tomography-Derived Extracellular Volume in TAVR Patients With Low-Flow Low-Gradient Aortic Stenosis. JACC Cardiovascular Imaging 2020 Oct 28
  - Scully PR, et al. Identifying Cardiac Amyloid in Aortic Stenosis: ECV Quantification by CT in TAVR Patients. JACC Cardiovascular Imaging 2020 Oct 13.

Minor comments

**Comment**: *Abstract. Similar sentences are repeated for the result and conclusion in the abstract. The presentation of the abstract should be more organized*

**Response**: We performed some changes in the abstract to make it more clear and organized.

**Comment:** **"***Introduction. Second paragraph. “Another MRI technique used to quantify DIF is T1-mapping, yet, its key limitation is limited spatial resolution”. The authors put too much emphasis on spatial resolution. At a lower concentration of contrast media, contrast discrimination by X-ray CT is not as good as T1 mapping CMR".*

**Response**: We removed the text about T1 mapping from the introduction, based on the comment from the same reviewer (please see our previous response).

**Comment: "***Method Study cohort. Please clarify that 10 of 85 AS patients were evaluated due to inadequate CT image quality. Was a prospective study, or a retrospective study that analyzed As patients who had sufficient CT image quality?"*

**Response**: We thank the reviewer for this comment. We re-phrased the "study cohort" section, and we believe it is clearer now (**Methods and materials section, study cohort subsection, page 5, 2^nd^ paragraph**).

**Comment**: "*CT data acquisition and reconstruction. “Besides the pre-contrast scan, a unique post-contrast scan with the same scan parameters was added”. Delete unique. Please add a brief explanation as to why post-contrast images were acquired at 7 minutes".*

**Response**: We thank the reviewer for this comment. As requested, the word unique was substituted with "additional". The accumulation of contrast agent in the interstitial tissue needs about 7 minutes as has been used by many previous publications (For example Abadia AF et al. J. Cardiovasc comput tomogr. 2020; 14: 162-167).

**Comment**: "*Result. Table 1. There was a significant difference in age between AS patients and control subjects (57.2±5.6 years in control subjects vs. 80.6±6.8 years in AS patients). Previous studies demonstrated that ECV was significantly influenced by age. In addition, prevalence of CAD was significantly higher in the AS group (p=0.003). The differences in age and the degree of diffuse atherosclerosis substantially influenced the ECV.*

**Response**: We thank the reviewer for this comment. Indeed, the AS group patients were older and had more comorbidities. There is no doubt that some of the difference in ECV between the groups might be attributed to this age difference. However, our data suggests that the AS itself is a major cause of myocardial fibrosis too. We also performed a multivariate analysis using linear regression, which showed that ECV fraction was significantly higher in patients with AS compared to the control group, independent from age, gender, BMI, diabetes mellitus, and hypertension (**this appears in the results section, page 13, 1^st^ paragraph).**

We add a table that describes this multivariate analysis. Because our paper has many figures already, it was not added to the paper. Should the reviewer wants us to add this table to the manuscript, we will be happy to do so.

Multivariate analysis for comorbidities between the AS group and the control group

| **Adjusted factor** | **Estimate** | **Std. Error** | **P value** |
| --- | --- | --- | --- |
| Aortic stenosis | 22.19 | 4.91 | <0.0001 |
| Age | 0.27 | 0.17 | 0.1 |
| Gender | 1.61 | 2.12 | 0.4 |
| BMI | 0.12 | 0.22 | 0.5 |
| DM | -3.53 | 2.36 | 0.1 |
| HTN | 0.62 | 2.72 | 0.8 |

BMI: body mass index; DM: diabetes mellitus; HTN; hypertension

**Comment**: "*In this paragraph, the authors also stated that multivariate analysis showed that ECV fraction was significantly higher in patients with AS compared to the control group, independent from age, gender, BMI, diabetes mellitus, and hypertension. However, the number of patients was limited to adequately confirm the independency of these parameters.*

**Response**: Although the number of the included patients were limited, the results were still significant according to our analysis. Please see the table attached for the previous comment.

**Comment**: "*Results. Figure 6B. NYHA FC at 12 months is a categorial parameter and a scatter plot is not suited for Figure 6B".*

**Response**: We fully agree with the reviewer, but some patients have a NYHA FC between 2 and 3 for example, therefore we thought that a scatter plot would be more appropriate.

**Comment**: "*Results. Figure 7. Comparison between the highest decile of ECV and lowest ductile of ECV seems to be arbitral".*

**Response**: We thank the reviewer for this comment. This figure represents the upper and lower ends of ECV. The message we want to convey, is that it is possible that Gal-3 levels might be associated with ECV fraction. We believe we could not demonstrate a clear relationship between ECV and Gal-3 in our entire cohort because of our low number of patients. This difference which exists only in the upper and lower deciles clearly does not mean that the relationship exists, it is merely suggesting it, as we stated in the results

**Comment**: "*Page 12. “There were no deaths.” Delete all-cause mortality from the combined endpoints to avoid confusion by the readers".*

**Response**: We thank the reviewer for this comment. We agree that since there were no deaths in our study, the outcome of all-cause mortality seems irrelevant. Therefore, we deleted it from our combined outcome.

**Comment**: "*Results. Figure 8. ROC curve. Add cut-off value for ECV"*.

**Response**: We thank the reviewer for this comment. Optimal cut-off value. Sensitivity/specificity decision plots yielded an ECV fraction of 40.8% as the optimal cutoff points for prediction of clinical outcomes, with a sensitivity of 91% and specificity of 64%. Patients with an ECV fraction >47.8% had the highest likelihood for the combined clinical outcomes of stroke and hospitalization for heart failure at 12-months, compared to patients with an ECV fraction < 30.5%, (31.6% vs. 10.1%, p=0.02). **This text appears in the results section, page 13, 2^nd^ paragraph.**

**Comment**: "*Page 14. Discussion. “Patients in the highest deciles of ECV fraction had a significantly higher serum Gal-3 level than patients in the lowest deciles. The combination of two different methods for assessing and quantifying cardiac damage (ECV and Gal-3) enforced our findings”. Comparison between the highest decile of and lowest ductile was quite subjective and this statement had no sufficient rationale*".

**Response**: We thank the reviewer for this comment. We erased the term "enforces" and rephrased that this finding "suggests that ECV fraction could be an important factor…" **(Discussion section, page 17, 1^st^ paragraph**).

**Reviewer 3:**

Thank you for your helpful comments and suggestions. Below please find the responses that were incorporated into the manuscript.

**Comment**: "*CTecv was calculated in the septum pre and post contrast as perviously described in CMR and CT manuscripts. As it is supposed to reflect diffuse myocardial fibrosis, the basal septum should be representative as it is relatively spared from myocardial infarction. With that in mind, I am very concerned about the distribution of the CTecv with 11 patients having an ECV >50% and a majority an ECV >40%. This amount of interstitial expansion is rather unusual unless there is evidence of myocardial infiltation (e.g. cardiac amyloidosis). The alternative is a methodological error".*

**Response**:  Only 6 patients in the entire group had previous MI and care was taken to avoid ECV measurement in segments with previous MI. In fact, the mean ECV in 6 patients with previous MI was only 37%. Since the basal septum is close to the membranous septum, we decided to use the mid septum for ECV calculation, as performed in previous studies (For example Bandula et al. 2013; 269). The increased ECV value might be related to the AS severity and could be in part the explanation of the high ECV value in our study population. But we have to mention, that the coexistence of cardiac amyloidosis in our study population cannot be fully excluded, this was stated in the study limitation (page 16, line 342-344).

**Comment**: "*The abstract should include structured headings, and the authors should include a heading for the discussion, which is missing".*

**Response**: We thank the reviewer for this comment. Those mistakes were corrected.

**Comment**: "*The authors have chosen Galactin-3 rather than established cardiac biomarkers like BNP or troponin in this paper. Although this is interesting, the lack of BNP and troponin feels like an oversight".*

**Response**: We thank the reviewer for this comment. Indeed, serum BNP and troponin are important biomarkers in cardiac patients. When designing this trial, we though the most novel and interesting marker to be tested is galectin-3, which was shown to reflect fibrotic processes across the human body. In this trial we did not measure BNP and troponin, and we do not have their levels for our patients, since they are not routinely taken before TAVI/AVR.

**Comment**: "*The combined clinical endpoint of all-cause mortality, stroke and hospitalization for heart failure is misleading and not ideal: -- although all cause mortality and HHF are adequate endpoints, the link between diffuse fibrosis and stroke is not clear. -- no deaths occured, only HHF and stroke -- the highest quartile of ECV chosen here was ECV>47.8% which is unusually high, and can only be explained by amyloidosis (see above)".*

**Response**: We thank the reviewer for this comment. It is indeed not clear whether this is an incidental finding or not. We added the sentence "The demonstrated relation between stroke and diffuse myocardial fibrosis is not clear at this moment, however it might be related to the fact that of the 3 patients who had a stroke, 2 had atrial fibrillation. The existence of atrial fibrillation in those patients might suggest a possible higher degree of myocardial fibrosis compared to patients without atrial fibrillation" to the discussion section **(page 16, last paragraph).** As we mentioned previously, we cannot exclude the coexistence of cardiac amyloidosis, however, the increased value of ECV could also be related to the severity of AS and accordingly to the outcomes in this patients.

**Comment**: "*Regarding endpoints, it is unclear how many endpoints occured periprocedural".*

**Response**: We thank the reviewer for this comment. In our cohort there were no periprocedural deaths. Among 3 patients who suffered a stroke – one had the event 6 months post procedure, and 2 within the first 6 months after the procedure. None of the events was peri-procedural.

 
Specific Comments.

**Comment**: "*The authors need to make it clear in the abstract that on 57/75 patients underwent aortic valve intervention (and what intervention TAVR vs SAVR vs valvuloplasty)".*

**Response:** the phrase "Out of the 75 patients in the AS group, 49 underwent TAVI, six surgical AVR, two balloon valvuloplasty, and 18 did not undergo any type of intervention". Was added to the abstract.

**Comment**: "*Introduction: The CMR section does not mention ECV quantification (just T1 mapping, which is used for ECV calculation) - this is mentioned in the first paragraph without mentioning the associated imaging modalities (CMR and CT)".*

**Response**: Based on the comments of reviewer 2, we skipped T1 mapping from the introduction, since it is not important for the focus of our study. We fully agree with the reviewer that pre-contrast and post-contrast T1 mapping are in parallel with the method used in our study.

**Comment**: *The technological improvements in CT are for certain, though relevant to ECV quantification is not temporal resolution, but Hounsfield unit stability as well as signal to noise ratio.*

**Response**: We thank the reviewer for the comment and fully agree with it, therefore the importance of temporal resolution was skipped from the introduction, as has also been mentioned by reviewer 2.

**Comment**: *When discussing CMR predictors of outcome in AS, please add ECV by CMR (Everett et al JACC 2020), which is probably more relevant than the LGE papers.*

**Response** – As requested, this reference was added.

Methods: 
**Comment**: *Please specify ethics committee reference and the period of recruitment.*

**Response**: The study was approved by the ethics committee as we mention that in page 6, line 3^rd^ paragraph. We added the recruitment period "between 2016 and 2018" in page 5, study cohort subsection.

**Comment**: *please specify which contrast agent you administered*.

**Response**: nonionic contrast agent (Iopromide 370; Bayer Schering, Berlin, Germany) was used, we added it in page 7, 1^st^ paragraph.

**Comment**: *please provide more details for the control cohort. Did this cohort undergo CT for a clinical indication or purely for research? This cohort is neither age nor sex matched, and have significantly less hypertension, CAD and renal impairment than the AS cohort.*

**Response**: The control cohort included patients who were admitted to the hospital for chest pain, and were referred for CT angiography for the purpose of ruling out coronary disease. Before patients were sent to the CT scan, they had to give their consent for an additional post contrast scan that was performed immediately after the routine scan was done. All patients signed an informed consent. This explanation was added to the methods section (**Materials and methods, study cohort subsection, page 5)**.

Indeed, the control cohort is not matched to the study group, obviously because AS patients are very old and tend to have co-morbidities. The purpose of creating this control group was to present the difference in ECV between AS patients and healthy individuals. There is no doubt that some of the difference in ECV between the groups might be attributed to this age difference and possibly to other comorbidities. However, our data suggests that the AS itself is a major cause of myocardial fibrosis too. We also performed a multivariate analysis using linear regression, which showed that ECV fraction was significantly higher in patients with AS compared to the control group, independent from age, gender, BMI, diabetes mellitus, and hypertension (**this appears in the results section, page 13, 1^st^ paragraph).**

We add a table that describes this multivariate analysis. Because our paper has many figures already, it was not added to the paper. Should the reviewer wants us to add this table to the manuscript, we will be happy to do so.

Multivariate analysis for comorbidities between the AS group and the control group

| **Adjusted factor** | **Estimate** | **Std. Error** | **P value** |
| --- | --- | --- | --- |
| Aortic stenosis | 22.19 | 4.91 | <0.0001 |
| Age | 0.27 | 0.17 | 0.1 |
| Gender | 1.61 | 2.12 | 0.4 |
| BMI | 0.12 | 0.22 | 0.5 |
| DM | -3.53 | 2.36 | 0.1 |
| HTN | 0.62 | 2.72 | 0.8 |

BMI: body mass index; DM: diabetes mellitus; HTN; hypertension

**Results:**

**Comment**: "*Please restructure this and place the figure legends to the end of the manuscript.*

**Response**: According to the journal instructions, these legends should be incorporated in the text **("**Figure captions must be inserted in the text of the manuscript, immediately following the paragraph in which the figure is first cited (read order). Do not include captions as part of the figure files themselves or submit them in a separate document). We would be happy to change this upon request.

**Comment**: *how many patients had pacemaker or ICDs? These are more likely to affect myocardial HU then AoV Calcification*.

**Response**: While initially recruited 85 patients with symptomatic severe AS, 10 patients were excluded due to pacemakers, implantable cardioverter defibrillators, metallic foreign objects in the proximity of the heart, and surgical aortic valve replacement. All of these may lead to beam-hardening artifacts, which may degrade the inaccuracy in the ECV measurements. This sentence now appears in the manuscript (**Materials and methods section, study cohort subsection, page 4**)

**Discussion:**

**Comment**: *please add a heading and restructure the discussion to follow a logical flow.*

**Response**: The first paragraph was divided in 2, the first one summarizes the results of our study and the second one disscuss the importance of ECV evaluation. In addition, we added a separate paragraph for limitation.

**Comment**: *Please add reference and discuss papers on CTecv by Tamarapoo and Scully that address CTecv in TAVI and outcome (both JACC Imaging).*

**Response**: As requested, these papers were added and discussed..
