# Supplementary material for: Myocardial extracellular volume quantification by computed tomography predicts outcomes in patients with severe aortic stenosis
Source: PLoS One. 2021 Mar 10;16(3):e0248306. doi: 10.1371/journal.pone.0248306 (PMC7946277; doi:10.1371/journal.pone.0248306)
Supplement: Attachment — Submitted filename: response to reviewers4.docx [file pone.0248306.s003.docx]

**Reviewer 1:**

Thank you for your helpful comments and suggestions. Below please find the responses that were incorporated into the manuscript.

**Comment: "** *A scatterplot is not appropriate for figure 6b - please change - better would be a boxplot as NYHA is definitely categorical.*"

**Response**: As requested, The correlation between ECV fraction and NYHA class at 12 months is now represented via a boxplot graph. Correlation was measured via a spearman correlation test. **See figure 6b**

**Comment: "** *The lack of troponin and/or BNP needs to be at least mentioned in the limitations - given their use it should be at least discussed as galectin is measured.*"

**Response**: As requested, this issue is now mentioned in the limitation paragraph (**page 16, limitation paragraph**).

**Comment: "** *The variability in ECV should also be mentioned. The authors should at least note that they do have some high ECV values. They mention that they cannot exclude amyloidosis in the limitations, and they should add that in the context of the identified high ECV values this could be relevant.*"

**Response**: As requested, this issue is now mentioned in the limitation paragraph (**page 16, limitation paragraph**).

**Comment**: " *The authors should also add in the limitations that the cohort was not matched for age or other important comorbidities such as hypertension"*

**Response**: As requested, this issue is now mentioned in the limitation paragraph (**page 16, limitation paragraph**).

**Comment**: " *The final limitation to add is that ECV was only taken from one site rather than a global measure or average, so it is possible that it may not be completely representative of the whole myocardium (accepting that this is the method used in most studies)".*

**Response**: As requested, this issue is now mentioned in the limitation paragraph (**page 16, limitation paragraph**).
